# Is environmental setting associated with the intensity and duration of children's physical activity? Findings from the SPEEDY GPS study

**DOI:** 10.1016/j.healthplace.2012.11.008

**Published:** 2013-03

**Authors:** Emma Coombes, Esther van Sluijs, Andy Jones

**Affiliations:** a School of Environmental Sciences, University of East Anglia, Norwich, Norfolk, NR4 7 TJ, UK; b MRC Epidemiology Unit, Institute of Metabolic Science, Box 285, Addenbrooke's Hospital, Hills Road, Cambridge CB2 0QQ, UK; c UKCRC Centre for Diet and Activity Research, Institute of Public Health, Cambridge CB2 0SR, UK

**Keywords:** GPS, Physical activity, Bout, Intensity, Environment

## Abstract

Using a sample of English school children, we use accelerometery and global positioning systems to identify whether different intensities of activity (light, moderate, and vigorous) occur in different environments, and whether environments for bouts of moderate to vigorous activity (MVPA) vary from those for non-bout MVPA. We find that land uses such as buildings and roads and pavements were generally used for light activity, whilst green environments such as gardens, parks, grassland and farmland appear supportive of vigorous activity. Built land uses such as hard surface play areas were particularly used for activity of short duration. Future work may consider differentiating light activity from moderate and vigorous, and separating bout and non-bout MVPA to better identify environmental supportiveness for activity in children.

## 1 Introduction

There is evidence that characteristics of the physical environment around homes and schools may influence children's physical activity (PA) levels (de Vet et al., 2011), and a number of recent studies have combined Global Positioning Systems (GPS) and accelerometery with Geographical Information Systems (GIS) databases to gain novel insights into the environments children use for PA. Activity behaviours at school within the playground (e.g. Duncan et al., 2009; Fjørtoft et al., 2009) and outside school-time (e.g. Jones et al., 2009; Quigg et al., 2010; Wheeler et al., 2010) have both been studied. In all these studies, children were determined to be ‘physically active' when undertaking moderate to vigorous intensity physical activity (MVPA). No previous studies have examined light intensity activity, which is thought to still confer health benefits (Kwon et al., 2011). Where some studies measured total MVPA (e.g. Wheeler et al., 2010), others measured bouts where the child is continuously active over several minutes (e.g. Jones et al., 2009). However, whether the environments used for bout and non-bout activities differ is unclear. This may be important since non-bout activities include sporadic or intermittent PA, such as unstructured play in a park with friends, and are an important contributor to children's overall PA (Wickel and Eisenmann, 2007). In contrast MVPA bouts represent sustained activity, such as walking trips or participation in organised sports such as football, and have been shown to provide benefits on adiposity status that are greater than those that would be provided by the equivalent number of minutes of non-bout MVPA (Mark and Janssen, 2009).

Although there is some evidence that different land uses support different intensities of children's walking (Mackett et al., 2007), knowledge on the intensity of PA undertaken in different environments, as well as variations in environments used for bout and non-bout MVPA is lacking. Using data from the UK SPEEDY study, we build on our previous analysis (Jones et al., 2009) by investigating these two issues.

## 2 Methods

We used a sample of 100 children aged 9–10 years old recruited from the SPEEDY (Sport, Physical activity and Eating behaviour: Environmental Determinants in Young people) cohort. The design of the SPEEDY study is described in Van Sluijs et al. (2008) and details of the 100 children selected for this analysis are described in Jones et al. (2009). Briefly, children wore an ActiGraph GT1M accelerometer and a Garmin Forerunner 205 GPS unit over four consecutive days, including two weekend days. Data collection took place between July and October 2007 outside of school term time (either during the summer vacation or during the October half-term) in order to capture children's PA behaviours during their free time. The accelerometer was set at a 5 second epoch, whereas the GPS used an adaptive setting to preserve memory, where the frequency of recording was associated with the frequency with which the wearer changed speed or direction and resulted in a point being recorded every 1–10 s. Accelerometery data points were matched to the closest recorded GPS location based on their date and time-stamps. Matching was only made if the time difference between the two sets of points was <30 s. Periods longer than this were coded as ‘missing’ because the child might have moved to a new unrecorded location. Matched data points were then classified into four intensity categories: sedentary [≤100 counts per minute (CPM)], light (101–1999 CPM), moderate (2000–3999 CPM), or vigorous activity (≥4000 CPM) (Ekelund et al., 2004). Bouts were identified and were defined as a period in which a child engaged in MVPA for 5 min, allowing up to 30% of bout time to be below this intensity. The bout length was determined based on previous work, which has demonstrated that sustained MVPA of at least 5 min confers health benefits in children (e.g. Mark and Janssen, 2009).

Matched data points were entered into the GIS package ArcGIS 9.2 and overlaid with a land use dataset developed using Ordnance Survey MasterMap (Ordnance Survey, 2012) and Centre for Ecology and Hydrology Land Cover Map of Great Britain datasets (Centre for Ecology and Hydrology, 2012). Together these databases provided information on 41 land use classes, and for analysis these were amalgamated. For example, we combined information on different types of woodland such as deciduous and coniferous into a single “woodland” category. This process gave us nine final land use categories; building locations, areas of other built land (including car parks and hard surface play areas), roads and pavements, domestic gardens, parks, farmland, grassland, woodland, and beaches. These land categories were selected based on evidence from previous studies that have demonstrated a broad range of land use characteristics are associated with PA levels including building density (de Vries et al., 2007), presence of parks and trees (Roemmich et al., 2006), and access to the coast (Bauman et al., 1999). Most studies have focused on urban settings, and given that our sample included children living in rural locations, we also incorporated into our database land uses that are prevalent in this setting such as farmland and grassland. Each GPS data point was assigned a land use category based on the land parcel it fell within.

We calculated the percentage of recorded time that children spent undertaking light, moderate, vigorous, bout MVPA, and non-bout MVPA in each land use. In addition to analysing the combined data we stratified our analyses by gender and whether the child's home was in an urban or rural location, where urban–rural status was identified using the Department for Environment, Food and Rural Affairs classification of settlements (DEFRA, 2012), as patterns of land use were found to differ between these factors in previous work (Jones et al., 2009). Analyses of variance were computed to test if the pattern of the percentage of time spent in different activity intensities differed between the land uses, and *T*-tests were undertaken to test these same differences between bout vs non-bout MVPA. Due to the small sample size (*n*=100), we focus on effect size as well as statistical significance when considering our findings. Statistical analyses were undertaken in SPSS v16.

## 3 Results

Table 1 shows the per capita mean daily minutes spent in different intensities of activity. On average, boys undertook more PA than girls at all intensities. Both boys and girls spent around 60% of recorded time in gardens and buildings.

**Fig. 1:**
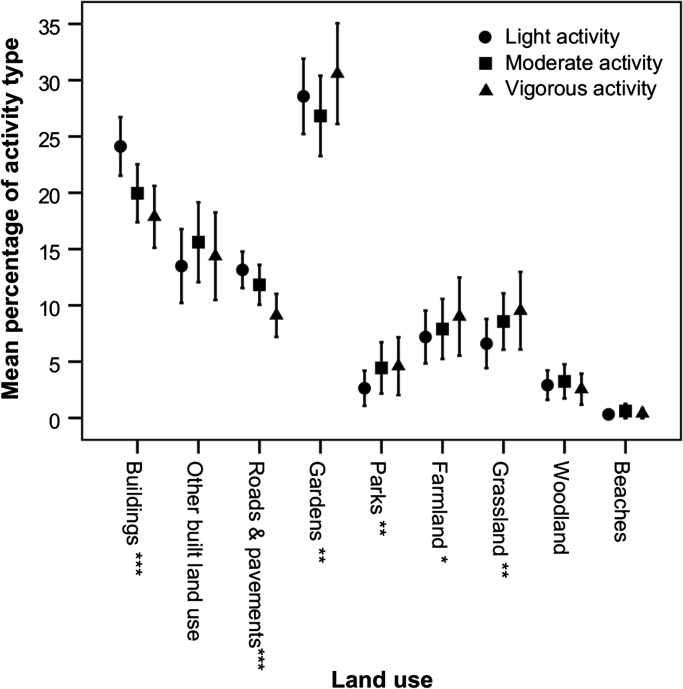
Mean percentage of light activity, moderate activity, and vigorous activity spent per child per day in each land use. Error bars are ±95% confidence intervals. Statistically significant difference between activity levels at *p<0.1,**p<0.05, ***p<0.001.

There were significant differences between the percentage of each child's light, moderate, and vigorous activity that was undertaken within each land use (Fig. 1). A significantly greater percentage of light activity (24.1%) compared to moderate (20.0%) and vigorous (17.9%) was undertaken in buildings (*p*<0.001) equating to an average of around 15 min more per child per day, and on roads and pavements (13.2% vs 11.8% and 9.1%, *p*<0.001) equating to around 7 min more per day. In contrast, a significantly greater percentage of vigorous activity (30.6%) was undertaken in domestic gardens compared to light (28.6%) and moderate (26.8%) activity (*p*=0.009), although the actual number of minutes spent in vigorous activity in this setting (4 min per day) was less than light (26 min per day) and moderate (7 min per day), reflecting the fact that overall less time is spent in vigorous activity. Similarly a significantly greater percentage of vigorous activity was undertaken in parks (*p*=0.011) and grassland (*p*=0.005), whilst borderline significance was found for farmland (*p*=0.075). This suggests that these ‘green environments’ may be especially supportive of higher intensity PA, although the actual number of minutes spent in vigorous activity in each of these environments is again small (<1.5 min per day).

**Fig. 2:**
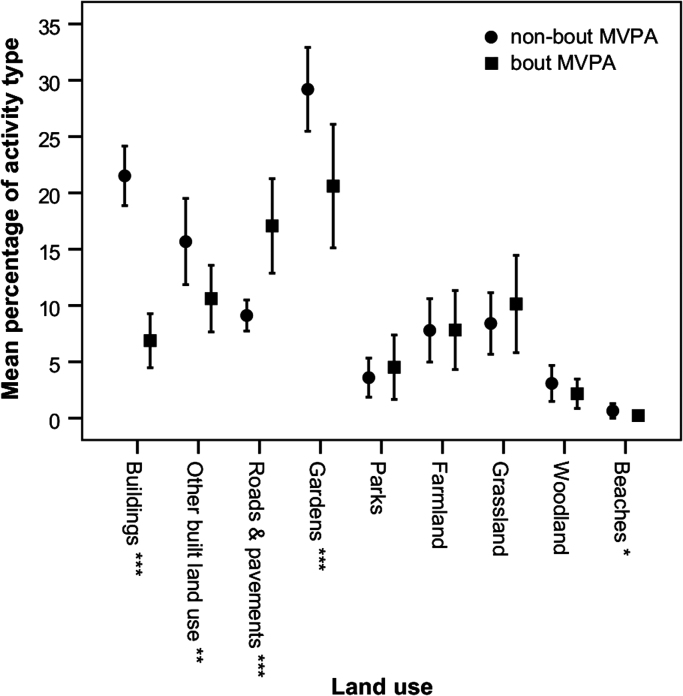
Mean percentage of bout MVPA and non-bout MVPA spent per child per day in each land use. Error bars are ±95% confidence intervals. Statistically significant difference between bout and non-bout at *p<0.1,**p<0.05, ***p<0.001.

Differences in the percentage contributions of land uses to MVPA were observed when bout and non-bout activity was compared (Fig. 2). A significantly greater percentage of non-bout activity was undertaken in buildings (*p*<0.001, 21.5% vs 6.9%), equating to 5 min more per day; other built land use (*p*=0.015, 15.7% vs 10.6%), equating to 3 min more per day; domestic gardens (*p*<0.001, 29.2% vs 20.6%), equating to 6 min more per day, whilst borderline significance was found for beaches (*p*=0.070, 0.6% vs 0.2%) equating to just 6 s difference reflecting the fact that beaches were not visited regularly. In contrast, a significantly greater percentage of bout activity was undertaken on roads and pavements compared to non-bout activity (*p*<0.001, 17.1% vs 9.1%), although this equates to just 36 s difference because of the small amount of time spent in bouts. Stratification by gender and urban-rural status did not substantially modify these findings as there were no noteworthy differences in terms of statistical significance or effect size (results not presented).

## 4 Discussion

The purpose of this study was to examine the manner by which children undertake different intensities and lengths of activity in different environments. In terms of activity intensity, buildings and roads and pavements were used relatively more for light activity. For buildings this is likely to be because they do not provide sufficient open space for children to undertake sustained higher intensity PA. This finding is consistent with Cooper et al. (2010) who found that the amount of PA undertaken outdoors was 2–3 times higher than that undertaken indoors in primary school children. Roads and pavements also appear supportive of bouts of MVPA, possibly reflecting the fact that children use them for walking trips, which generally consist of more sustained activity. Notably, Mackett et al. (2007) found that children walked faster and more intensely on roads and pavements when accompanied by an adult, although we had no information here on whether the children were accompanied. In comparison, green environments including domestic gardens, parks, grassland, and farmland appear more supportive of vigorous activity, reflecting the fact that they provide more open space and, therefore, opportunity for intense PA such as active play and informal sports. In line with previous studies (e.g. Quigg et al., 2010; Wheeler et al., 2010) we found that PA in green environments only accounts for a small proportion of the total time that children spend active outdoors, yet this activity makes an important contribution to overall PA since the activity undertaken in green environments is at a higher intensity than that in non-green environments. In terms of activity length, buildings, other built land uses, gardens, and beaches were used more for non-bout MVPA, probably reflecting their importance for unstructured play. In particular the often small size of modern domestic gardens may limit their supportiveness for longer bouts of activity.

Overall, in terms of the absolute number of minutes of PA undertaken in each of the land uses, the differences between the most and least used environments were small. For example, there was only 7 min difference comparing the mean time per day spent undertaking moderate PA in gardens, the most used environment, to beaches, the least used environment. For vigorous activity, the difference was even smaller, just 4 min. These small differences in absolute values reflect the fact that children are only active at higher intensities for a relatively small proportion of the day. UK guidelines recommend that children undertake 60 min of MVPA per day (Department for Health, 2011), and previous studies have demonstrated that 45 min of moderate PA and 15 min of vigorous PA are associated with reduced body fat and BMI (e.g. Wittmeier et al., 2008). Based on our analysis the contribution made by some of the land uses in terms of helping children achieve these targets is relatively small.

Study strengths include data collection outside the school term, when children were less restricted in their ability to use different environments, although an implication of this is that some of the environments used, such as beaches and woodland, may be more dominant than during other times of the year. Norfolk is also environmentally heterogeneous, and the sample was chosen to maximise this heterogeneity, containing children living across the urban–rural spectrum. Limitations include the small sample size, and that GPS devices were often removed when participating in team sports and swimming. It is, therefore, likely that the types of environment such as parks, in which activities such as soccer are commonly played, are underrepresented. Furthermore, accelerometers are known to poorly measure activity levels during cycling (Sirard and Russell, 2001) and the intensity of this behaviour is likely to have been underrepresented. Due to the difficultly of defining when a bout ends, given that some time is generally allowed to drop below the chosen PA intensity threshold, it can be difficult to capture all bout activity a child undertakes. We defined bouts as being blocks of 5 min in length, and a limitation of this is that we may have recorded some bout activity as non-bout. Finally, the GPS devices we used recorded locations with a spatial accuracy of approximately 3 m when in clear view of satellites, but GPS can return inaccurate locations when the signal is poor. Whilst we visually checked data points for obvious errors, it is likely that some misclassification is still present, although it is likely to be non-differential.

In conclusion, our findings in this sample of children show differences in the environments used for activity intensity and length. We recommend that future work seeking to identify the role of the environment in supporting PA should differentiate light activity from moderate and vigorous, and separate bout and non-bout MVPA to better identify the extent to which different environments support varying intensities and lengths of activity. Differentiation of activity length is particularly important in children amongst whom unstructured play means that non-bout based activities make a much greater contribution to overall PA than in adults (Mark and Janssen, 2009).

## Figures and Tables

**Table 1:** Mean number of minutes spent undertaking different intensities of activity per child per day and the mean number of minutes spent in different land use types, by gender. Standard deviations (SD) are given in brackets.

Measure	**Boys** (*n*=47), mean (SD)	**Girls** (*n*=53), mean (SD)	**Total** (*n*=100), mean (SD)
**Activity intensity (min)**			
Light activity	169.1 (35.9)	167.0 (33.4)	168.0 (34.4)
Moderate activity^⁎⁎^	46.5 (15.1)	38.3 (9.7)	42.2 (13.1)
Vigorous activity^⁎⁎^	24.1 (14.7)	16.5 (9.6)	20.2 (12.8)
Bout MVPA^⁎⁎^	19.8 (13.1)	10.9 (9.8)	15.1 (12.3)
Non-bout MVPA^⁎⁎^	50.8 (20.8)	43.9 (15.5)	47.3 (19.0)

**Land use (min)**			
Buildings	107.8 (70.0)	89.2 (67.8)	97.9 (69.1)
Other built land use	45.3 (68.8)	42.8 (62.8)	44.0 (65.3)
Roads and pavements^⁎⁎^	44.8 (24.6)	35.0 (24.7)	39.6 (25.0)
Gardens^⁎^	136.8 (98.3)	101.3 (78.4)	118.0 (89.6)
Parks	9.2 (17.6)	3.9 (11.6)	6.4 (14.9)
Farmland^⁎^	29.5 (52.2)	20.9 (56.0)	24.9 (54.2)
Grassland	24.2 (63.0)	22.5 (41.1)	23.3 (52.3)
Woodland	12.6 (29.6)	6.0 (11.7)	9.1 (22.1)
Beaches	0.6 (1.9)	0.7 (4.2)	0.6 (3.3)

Differences between boys and girls: ^⁎^*p*<0.1, ^⁎⁎^*p*<0.05.
